# Transport of Aerosols in Underground Mine Workings in Terms of SARS-CoV-2 Virus Threat

**DOI:** 10.3390/molecules26123501

**Published:** 2021-06-08

**Authors:** Krystian Skubacz, Robert Hildebrandt, Aleksandra Zgórska, Zdzisław Dyduch, Krzysztof Samolej, Adam Smolinski

**Affiliations:** 1 Silesian Centre for Environmental Radioactivity, Central Mining Institute, Plac Gwarkow 1, 40-166 Katowice, Poland; kskubacz@gig.eu (K.S.); ksamolej@gig.eu (K.S.); 2 Central Mining Institute, Experimental Mine Barbara, Plac Gwarkow 1, 40-166 Katowice, Poland; rhildebrandt@gig.eu (R.H.); zdyduch@gig.eu (Z.D.); 3 Department of Water Protection, Central Mining Institute, Plac Gwarkow 1, 40-166 Katowice, Poland; azgorska@gig.eu; 4 Central Mining Institute, Plac Gwarkow 1, 40-166 Katowice, Poland

**Keywords:** Coronavirus, SARS-CoV-2, Aerosol transport, Mining ventilation, Underground mines

## Abstract

This paper presents a method of implementation and the results of aerosol dispersion tests in underground mine workings. Numerous tests were carried out to determine the potential risk of SARS-CoV-2 coronavirus infection in the underground environment of the mines. The influence of selected parameters of mine air on the possibility and method of aerosol transmission through ventilation routes was experimentally determined in real conditions. The concentration of additional aerosols in the class of ultrafine and fine aerosols increased with the distance from the generator, while the concentration of coarse particles decreased. Assuming the consumption of the solution with which aerosols were generated, even at a small level of 1 cm^3^/min., the number of additional aerosols was several hundred particles in one cubic centimeter of air at a distance of 50–70 m from the generator. The concentration of ultrafine particles in the range of 40–20,000 nm increased from 122 particles/cm^3^ to 209 particles/cm^3^ at air temperature of 12 °C and relative humidity of 95–96%, and from 90 particles/cm^3^ to 243 particles/cm^3^ at air temperature of 17 °C and relative humidity of 76–82%, with the increasing distance from the generator (10 m to 50 m).

## 1. Introduction

The main route of SARS-CoV-2 infection is through respiratory droplets, according to Word Health Organization (WHO) data. Most often, the infection occurs as a result of direct or indirect contact with a person suffering from COVID-19, through contact with their secretion, which is a carrier of the virus, such as saliva and respiratory secretions (including respiratory droplets) that are released during coughing, sneezing, speaking, or singing [1,2,3,4]. Nevertheless, recently published works indicate that, in addition to contact and droplet spread, the transmission of SARS-CoV-2 via aerosols is highly probable under certain, favorable environmental conditions (e.g., indoor spaces, poor ventilation, long duration exposure to high concentrations of aerosols, etc.), causing different countries, associations, and organizations to review their guidance [5,6]. The role of aerosols in viral transmission was taken into account, inter alia, by the Centers for Diseases Control and Prevention (CDC). In a scientific brief, which was published in May 2021, the CDC confirmed that the principal model by which people are infected by SARS-CoV-2 is through exposure to respiratory fluids that carry the virus. Importantly, the inhalation of very fine respiratory droplets as well as aerosol particles was identified as one of three main routes of exposure [7]. It is an undeniable fact that aerosols, both larger and smaller (< 5 μm), being excreted spontaneously and formed by the evaporation of larger particles, are carriers of the virus. Furthermore, the smallest very fine droplets, as well as the aerosol particles that formed when these fine droplets rapidly dry, are small enough to remain suspended in the air from minutes to hours [7]. Generally, aerosols are objects in air of a size from about 0.002 to more than 100 μm, which are usually stable for at least a few seconds [8]. Therefore, the use of the term “droplets” has been reserved to objects that are generated immediately after coughing and sneezing that remain close to the source or objects that are much higher in size than 100 μm.

Viral infection via droplet takes place by an airborne secretion, in which viruses, with a diameter of virions in the 20–300 nm range, including SARS-CoV-2 with the dimensions of 40–200 nm, are present as infectious agents in the form of single particles, clusters, or are distributed on droplets or aerosols [9]. The Standing Committee on Emerging Infectious Diseases and 21st Century Health Threats (USA) has confirmed the possibility of spreading SARS-CoV-2 together with aerosols that are released with exhalation, coughing, or sneezing of a COVID-19 infected person [10]. Literature data proved that virus-containing body secretions can be aerosolized into infectious virus-containing droplets or particles in a variety of ways; moreover, respiratory secretions are known to be aerosolized through normal daily activities (e.g., talking, singing, exhaling, coughing, or sneezing) [6]. In terms of the latest research, several papers have highlighted the role of aerosol in the transmission of coronavirus SARS-CoV-2 by the airborne route [11,12,13,14,15]. Additionally, studies conducted after the SARS-CoV-1 epidemic demonstrated that airborne transmission was the most likely mechanism explaining the spatial pattern of infections. The retrospective analysis conducted for SARS-CoV-2 showed the same results [12]. Depending on their dimensions, the airborne particles settle on objects in the patient‘s environment or take the form of aerosols containing viruses, which, when inhaled together with the inhaled air, pose the risk of infection.

The literature data show that, during sneezing, the human body excretes approximately 40,000 drops (diameter of about 0.5–12 μm). Coughing generates approximately three-thousand drops, which corresponds to the number of drops exhaled during five minutes of speech [9]. The respiratory droplet size range is 1–500 μm, according to the literature data [9]. The size of the drops in the air that are exhaled by a healthy person range from 0.1–10μm. During speech, the droplet size is 0.1–12 μm on average, while, during coughing, droplets with a diameter of 0.1–16 μm comprise the vast majority [16]. In percentage terms, 62% of the drops that are present in the exhaled air have a diameter of < 12 μm (over 70% are between 2–20 μm). Similarly, in the case of coughing, drops with a diameter of < 12 μm represent over 72% of all emitted particles [17]. After the release of infectious droplets, their size and air flow are the main factors determining their movement. The scientific experiments that have been conducted so far have allowed, among others, to determine the percentage share of individual fractions of respiratory drops in the exhaled air, to estimate the variability of the drops excreted in the speech of a sick and healthy person, as well as to assess the impact of voice modulation on the number of respiratory drops generated [18,19,20]. However, the authors of the publication draw attention to the fact that, so far, no studies have been conducted for the infectious agent SARS-CoV-2 and the findings are drawn from experiments that do not reflect the actual environmental conditions and the amount of secretion released during speech and cough. The possibility of airborne virus transmission has been documented, i.e., by Booth et al. [21], Li et al. [22], Olsen et al. [23] or Yu et al. [24].

Additionally, recent papers also reported some quantitative viral RNA data. For example, research that was performed in a Singaporean hospital has proven the presence of the SARS-CoV-2 infectious virus in two out of three air samples from patient infection isolation rooms (1.8–3.4 viral RNA copies per liter air). A study conducted in the USA (Nebraska) confirmed the presence of the virus in more than 63% of tested air samples from various hospital rooms (mean 2,9 viral RNA copies per liter air) [25]. However, the studies that were carried out by the above-mentioned authors mostly refer to highly congested, and poorly or zero-ventilated, enclosed facilities/areas, such as: hospital rooms, conference rooms, and the interiors of means of transport. Additionally, the simulation studies and modelling results that have been described in the literature were carried out in windless conditions, which did not take the influence of air masses on the transmission of respiratory drops into account [16,17,26].

In this paper, research has been undertaken into the spread of aerosols in underground mine workings, in terms of the risk of transmission of infectious diseases in this specific work environment. The phenomenon of above-average number of SARS-CoV-2 virus infections confirmed among miners of hard coal mines in Poland within the Silesian agglomeration is the rationale for undertaking research works in this area. According to government data from 4th March to 1st July this year, 34,775 cases of the virus infection were recorded in Poland, of which, for the Silesian province (an area with one of the highest population density and industry concentration rates), the number of confirmed cases was 12,855. The discussed problem concerns large industrial plants where the dynamic growth of infections significantly increases the risk of epidemic development, not only in these plants, but also in the whole region.

The observed phenomenon may indicate a potential impact of the environment and the organization of underground work on the conditions for aerosol dispersion, which results in an increased risk of infection.

The above thesis is verified by the conclusions formulated at the end of the paper, which are based on the results of research and experiments that were conducted in the real conditions of an underground mine.

## 2. Materials and Methods

The measurements of size distributions of aerosols were made using two particle spectrometers. Both of the spectrometers are products of TSI Incorporated (Shoreview, MN, USA). The first one, the Scanning Mobility Particle Sizer (SMPS) spectrometer, is designed to examine smaller particles in the range from 10 to 1000 nm. However, this range can be changed by adjusting the flow rate. During the tests, the flow rate was 0.3 dm^3^/min., which allowed registering particles in the 15–711 nm range divided into 109 measurement channels. The SMPS spectrometer is a system consisting of three basic assemblies: Electrostatic Classifier (EC), Differential Mobility Analyzer (DMA), and Condensation Particle Counter (CPC). There is a single-stage impactor that is placed directly at the air inlet to the device to remove the particles that are outside the measuring range. Inside the EC panel, there is a column with a beta radiation source (Kr-85: β^+^) that ionizes the flowing air. Consequently, the aerosol particles quickly reach a state of charge equilibrium. By entering the DMA column, the aerosol-containing air is combined with a 10 times stronger stream of aerosol-cleaned air. This is a formation stream that maintains a laminar flow along the column axis. In the central part of the column, there is a negatively charged electrode that produces an electrostatic field. As a result, the trajectory of the positively charged particles is curved towards the column axis. If the curvature of the trajectory is appropriate, then the particles are directed to the hole that is located in the bottom of the column. During each measurement cycle, the voltage of the electrode is gradually changed, so that particles of different mobility (sizes) reach the hole and enter the last module of the CPC spectrometer. The CPC counter detects particles of a certain size by laser. Before reaching the vicinity of the laser system, the particles move in an area with high humidity and become enlarged to the size that allows their detection. The software of the spectrometer allows for taking the loss of aerosols inside the tubes along which the air moves into account. The losses depend on the temperature and pressure of the air and the length of the tubes, and they are particularly important for very small particles due to their relatively high diffusion coefficient.

The other Aerodynamic Particle Sizer (APS) spectrometer is designed to record larger particles. It operates within a fixed aerosol size range of 542–19,810 nm. The measuring range is divided into 51 channels. Air flows into the spectrometer at 5 dm^3^/min. However, the stream is separated directly behind the inlet. The stronger stream of approximately 4 dm^3^/min. is directed, after passing through the filters, to the measuring system. It connects there with air containing aerosols that contribute to their acceleration, which depends on the inertia of the particles and, as a result, on the time of passage between the two laser beams. This allows for the assessment of their aerodynamic size. The results that are obtained from spectrometers are combined with a non-linear equation based on particle density and aerodynamic shape factor [8,27]. Aerosols based on salt solution (0.7 g NaCl/dm^3^) were produced during the study. Therefore, the particle density was close to 1 g/cm^3^ (1.0001 g/cm^3^), and they are similar in shape to a sphere and, as a result, both sizes become equivalent.

**Figure 1:**
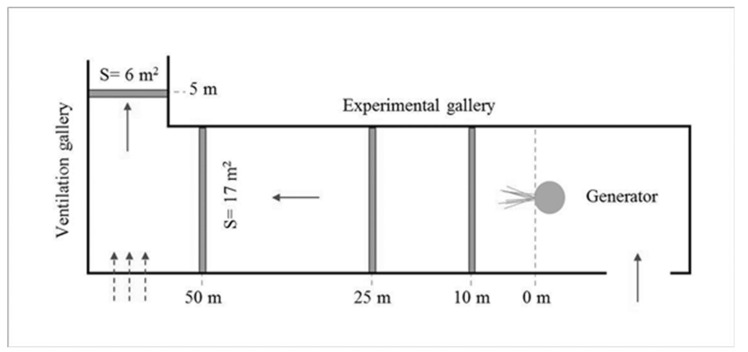
Diagram of the experimental gallery and arrangement of test stands and aerosol generator (S—excavation cross-section).

The research on aerosol dispersion was carried out in the Central Mining Institute (GIG)—Experimental Mine ‘Barbara’. Its underground workings infrastructure allows for conducting real scale tests, under real mining conditions. The test stands were prepared in the eastern part of the mine in the experimental working, called the experimental gallery, which is located 30m deep. Mining and ventilation conditions were the criteria for selecting this location, while the dimensions of excavations in coal mines and the possibility of regulating air flow in the area of works were mainly taken into account. The support of the galleries was made of steel arch support sets, and the lagging and the roof and side walls was by means of reinforced concrete slabs. The experimental gallery is characterized by the following dimensions: length L = 80 m, width w = 5.6 m, height h = 3.75 m, and cross-section along its entire length S = 17 m^2^. The ventilation gallery behind the intersection has the parameters L = 100 m, w = 2.7 m, h = 2.6 m, and S = 6 m^2^. Figure 1 schematically presents the area of the research.

**Figure 2:**
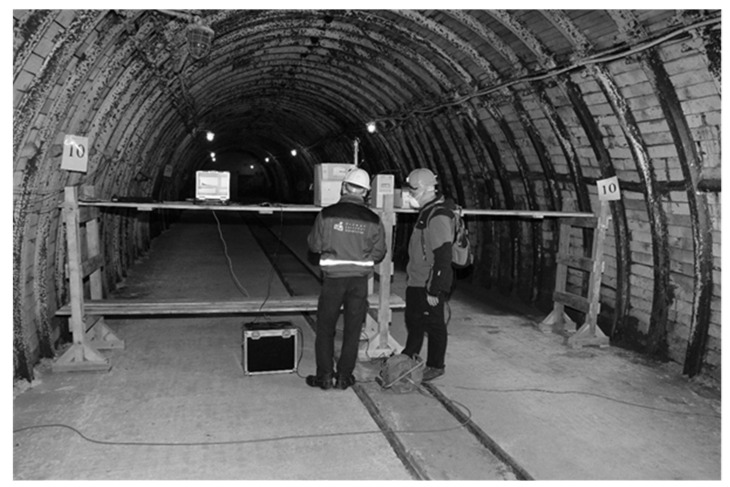
GIG Experimental Mine ‘Barbara’, level 30 m. The area of conducted research works and a view of the support structure for particle spectrometers.

The test stand is equipped with devices that ensure the continuous measurement of the ventilation parameters of mine air. The devices included anemometers, sensors of temperature, and sensors of humidity. The described measurement system cooperated with the surface telemetry unit where the acquired data were recorded. Apart from devices for microclimate monitoring, the experimental gallery contained an installation for aerosol production and portable height-adjustable platforms on which measuring equipment for scattered particles detection was placed (Figure 2). Devices ensuring a change of climatic conditions in the range of temperature and humidity of the flowing air were included as additional equipment for the area of the works; they were located outside the test stand.

**Figure 3:**
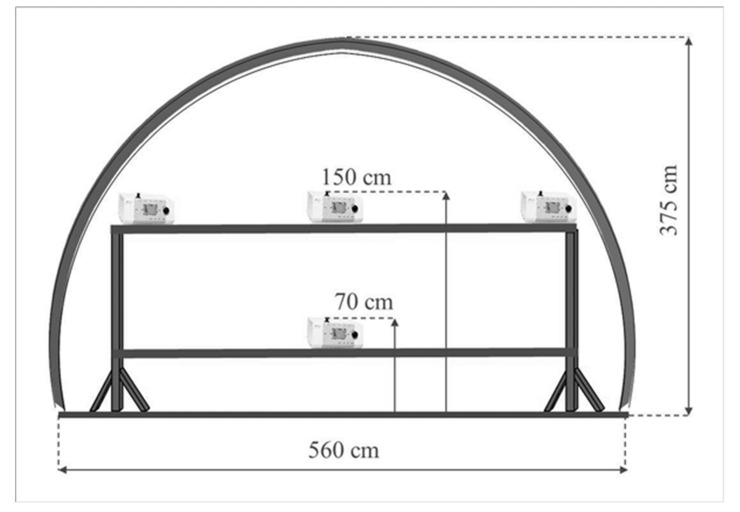
GIG Experimental Mine ‘Barbara’, level 30 m. The area of conducted research works and a view of the support structure for particle spectrometers.

During the tests, the speed of passing air in the experimental gallery was within the range of 0.4–0.7 m/s. The main fan of the mine installed on the surface at the ventilation shaft forced the airflow during the experimental working. Depression that is caused by the operation of this fan results in air movement towards the ventilation shaft and simultaneous fresh air inflow from the other shaft. It was also possible to adjust the fan’s thrust to regulate the air flow velocity. The experimental working was located approximately 200 m from the shaft supplying fresh air, and it provided stable (unchanged) conditions for conducting the research for both the background and generated aerosols. The air flowed in the direction of the intersection with the ventilation gallery, where its stream combined with an additional air current from behind the dam (approximately 15–20% by volume), and it reached a speed of 1.8–2.1 m/s. The test stands were located in the experimental gallery at distances of 10 m, 25 m, and 50 m from the aerosol generating nozzle (Figure 2). In each of these three positions, the aerosol size distributions were determined at the left and right side wall and in the excavation axis. The set of measuring devices was installed on the platforms, so that the air inlets to the spectrometers were at the height of about 150 cm, which was exactly the same height as the nozzle producing the aerosols and additionally at the height of about 70 cm from the floor in the axis of the excavation (Figure 3).

**Figure 4:**
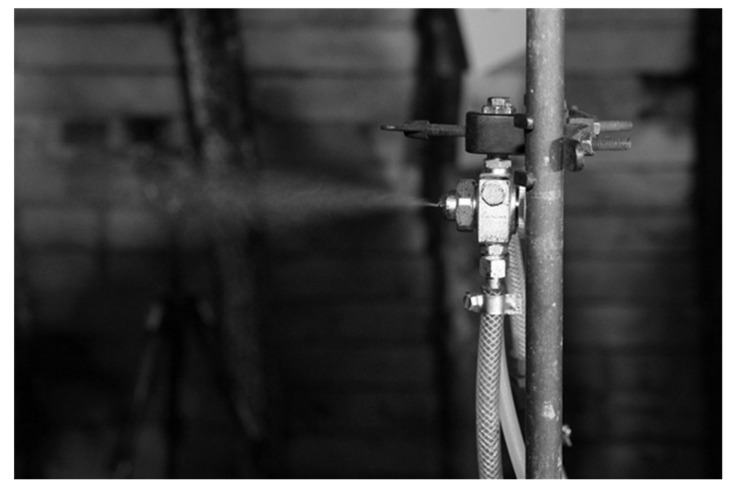
Nozzle during aerosol generation.

At each of these sites, the background was always measured immediately before aerosol generation. Subsequently, after switching on the generator, the aerosol size distribution was determined again three times in 3-min. cycles. The aerosol generator was still running during these measurements. Before starting the next cycle in another place, researchers waited some time to allow for ventilation of the excavation. The distance between the aerosol generator and measuring place caused a certain delay in the arrival of the ‘wave’ of aerosols. Therefore, when taking air velocity and distance into account, a certain amount of time has always been allowed to elapse until the ‘wave’ reaches the location of the particle spectrometers. With this procedure, the results of three consecutive measurements during aerosol generation did not differ significantly, and they were mainly caused by the irregular operation of the generator. The real solution consumption by the generator was monitored and noted at all times. In the same way, measurements were taken in the ventilation gallery, but, due to its small cross-section, only in the central part of the excavation at a height of about 150 cm. The aerosols were generated with the use of a Lumina ST-R type nozzle (Fuso Seiki, Co., Ltd, Tokyo, Japan) with an outlet diameter of 1.3 mm (Figure 4).

At a distance of 300 mm from the outlet, the nozzle produces an aerosol cone with a diameter of 95 mm. The Sauter mean diameter (SMD) is 44 µm at a distance of 200 mm. The required water pressure was obtained by placing a small water reservoir 0.8 m above the nozzle. The nozzle was fed with compressed air at a pressure of approx. 3 bar. The aerosol was made of water with a small addition of sodium chloride (0.7 g/dm^3^ of water). The solution was consumed at a rate of 7–20 cm^3^/min. The nozzle, with its outlet facing the air flow direction, was placed 150 cm above the floor, in the axis of the excavation.

The measurements were made in two different variants (Table 1). In the first case (variant A), the temperature was 12 °C and the relative humidity was between 89 and 95%. During the second measurement session (variant B), the temperature was increased using electric heaters, which were located approximately 10 m behind the aerosol generating station. Consequently, the temperature in the vicinity of this site reached 23 °C. However, it decreased gradually, reaching 16 °C at a distance of 50 m from the aerosol generating nozzle. At the same time, the relative humidity was much lower when compared to the previous conditions (Variant A). It oscillated between 50% and 82% as it moved away from the aerosol generator.

## 3. Results and Discussion

**Figure 5:**
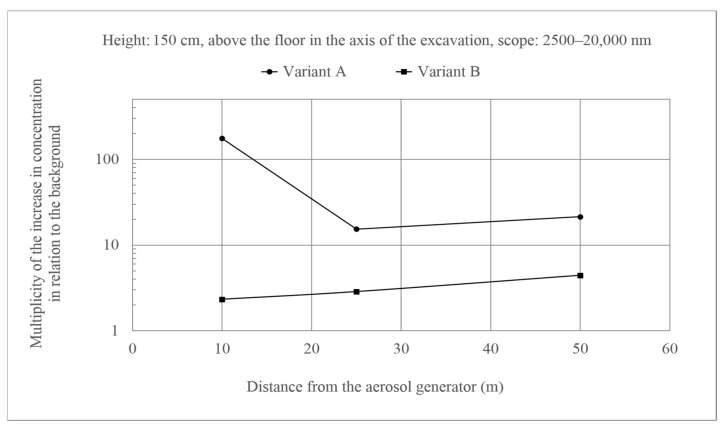
Increase of aerosol concentration of 2500–20,000 nm at the height of 150 cm above the floor in the axis of the excavation in relation to the background. Generator capacity: 17 cm^3^ of solution/min.

The additional aerosols that occur during nozzle operation undergo various transformations, which are determined by their own size distribution and concentration, size distribution and concentration of aerosols that are already present in the environment (ambient aerosols), and environmental conditions, such as temperature, pressure, and humidity. During transport, they can be removed from the air stream as a result of processes, such as diffusion, inertia, or gravitational sedimentation. The first of these mechanisms is relevant for small aerosols of a nanometer size, which are more mobile. Inertia, in turn, manifests itself when the particles are larger and move at a higher speed. When the air stream turns violently, such objects are unable to follow its direction and they hit the walls of the corridor along which the air moves. The particles are subjected to a gravitational force pulling the objects down, regardless of the air speed. This process becomes more important for large particles, which have a higher mass. Under the conditions of ventilated excavations, the concentration of additional aerosols is also reduced as a result of ventilation and the inflow of other independent air currents. Ultrafine aerosols are objects up to 100 nm, fine aerosols from 100 to 2500 nm, coarse aerosols from 2500 to 10,000 nm, and supercoarse aerosols that are above 10,000 nm. However, coarse aerosols have been attached to the class of coarse aerosols due to very small concentrations of supercoarse aerosols, and the given results refer to the extended range from 2500 to 20,000 nm (end of the spectrometer range). A very large increase in background concentration was observed for the largest aerosols at the stand that is located closest to the generator (Figure 5). At this point, the aerosols had not yet dispersed, and the environmental conditions during measurements at relatively low temperature and high humidity were not conducive to aerosol size reduction (Variant A). However, when the temperature was higher and the humidity was lower, the aerosols evaporated quickly, and the size of the aerosols declined (Variant B). In this case, the concentration of larger aerosols increased in relation to the background twice, and at higher humidity and lower temperature about 180 times at a generator capacity of 50 cm^3^ of solution/ 3 min. This means that, within about 30 s, taking the air velocity into account, the aerosols managed to reduce their size predominantly as a result of evaporation at higher temperatures and lower humidity (Variant B).

**Figure 6:**
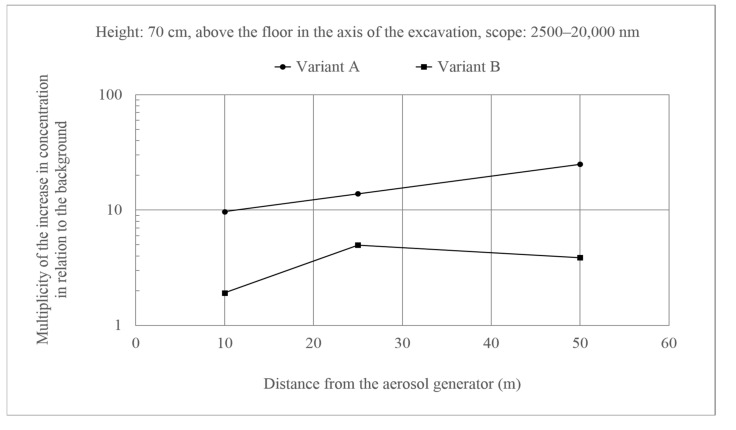
Increase in the concentration of aerosols with a size of 2500–20,000 nm at a height of 70 cm above the floor in the axis of the excavation as compared to the background. Generator capacity: 17 cm^3^ of solution/min.

Measurements that were taken at the height of 70 cm (Figure 6), where the background disturbance was much more pronounced for Variant A when compared to the observed concentrations for Variant B, also indicated the course of the process that is described above. However, it is worth noting the course of changes when there were more aerosols of larger sizes (Variant A). The concentration of particles at a distance of 50 m from the generator was significantly higher than at a distance of 10 m when compared to the background. This indicates a decrease in the flight trajectory of larger and heavier particles under the influence of gravity.

**Figure 7:**
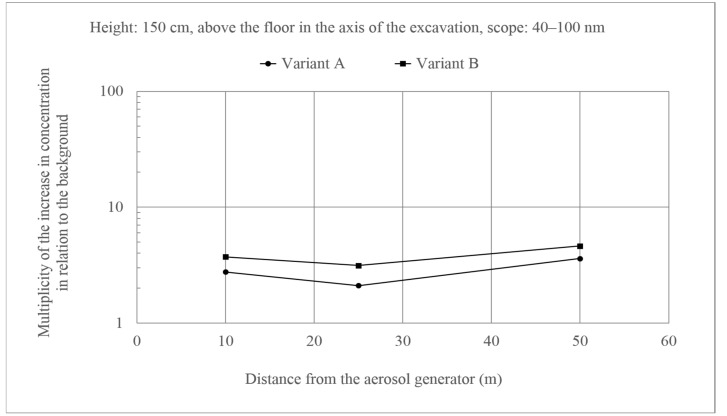
An increase in the concentration of aerosols of the size 40–100 nm at a height of 150 cm above the floor in the axis of the excavation in relation to the background. Generator capacity: 17 cm^3^ of solution/min.

**Figure 8:**
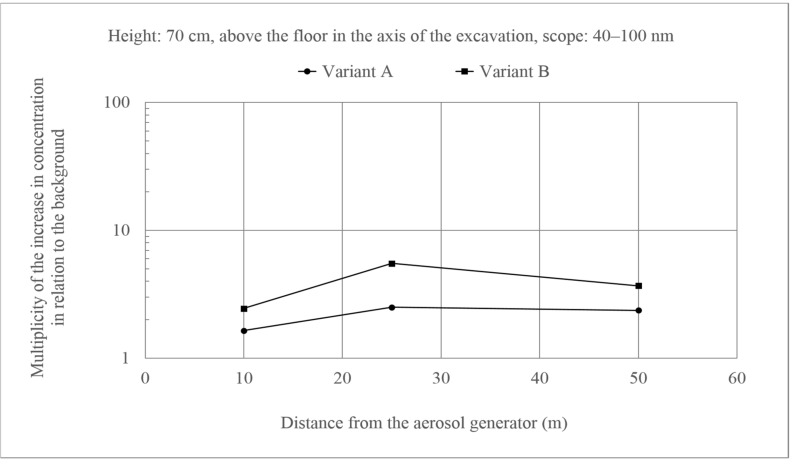
Increase in the concentration of aerosols of the size 40–100 nm at a height of 70 cm above the floor in the axis of the excavation in relation to the background. Generator capacity: 17 cm^3^ of solution/min.

Such effects were not observed for ultrafine particles (Figure 7 and Figure 8). In this case, the changes in concentrations in relation to the background were similar along the entire length of the excavation, and multiplicity of the increase in concentration in relation to the background did not exceed 10. There were also no differences between the two variants. Additionally, the behavior of these particles did not differ from the fine particles.

**Figure 9:**
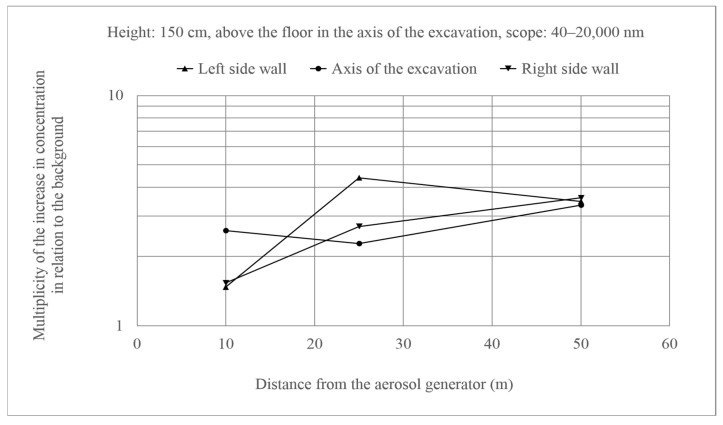
Increase in the concentration of aerosols of the size 40–100 nm at 150 cm above the floor in relation to the background. Generator efficiency: 17 cm^3^ solution/min (Variant A).

The greatest background disturbances were observed near the aerosol generator when analyzing the results obtained at a height of 150 cm at the left and right side wall and in the axis of the excavation, which is quite natural. From 25 m onwards, the differences began to decrease and, in the vicinity of the 50 m of the excavation, the background is already exceeded to the same extent for the left and right side wall and in the axis of the excavation. Figure 9 presents this mechanism of air mixing for all aerosols and what is noticeably a similar course was observed for ultrafine, fine, and coarse aerosols (Variant A).

Table 2 shows the concentration of the additional aerosol particles in unit volume per unit consumption of solution with a 1 cm^3^/cm^3^ generator. As the distance from the source of emission grows, the number of additional aerosols increases in the range of ultrafine and fine particles and at a distance. Such an increase could be due to the defragmentation of coarse aerosols or an increase in the size of aerosols that were smaller than the lower boundary of the measuring range. The analysis was specifically performed for sizes from 40 nm and above, because this is the minimum SARS-CoV-2 virus size. The lower measuring range of the SMPS spectrometer is 15 nm. However, the concentration increases in a similar way, even if the whole measuring range is considered. However, perhaps the aerosol generator also produced very small particles below 15 nm in size, on which condensation of water vapor took place during movement, which resulted in an increase in the ultrafine and fine particle class of the measurement range.

When assessing the possible transmission of the virus, not only is the concentration of the aerosols important, but also their volume. The concentration of coarse aerosols is small, but their volume is many times larger than the fractions that form the fine particle class, and even more so ultrafine. In Table 3, the results are presented in such an approach. It includes the volume of additional aerosols and the percentage share of particular classes. The proportions were reversed here, and the coarse particles show the largest percentage share in such an approach, as could be assumed. It decreases with an increasing distance from the aerosol generator. This decrease is more significant under conditions of lower humidity and higher temperature (Variant B). In this case, the proportion of fine particles becomes most significant at further distances. The volume of ultrafine particles remains consistently low, although it was much higher at higher temperatures and lower humidity. The total volume of additional aerosols at a lower temperature and higher humidity decreased at a distance of 50 m approximately twice when compared to measurements that were taken 10 m from the generator. On the other hand, during the measurements carried out at higher temperature and lower humidity (Variant B), it grew with increasing distance, but its absolute value was almost an order of magnitude lower when compared to the situation when the temperature was lower and relative humidity higher (Variant A).

The cross-section of the experimental gallery was about 17 m^2^. Therefore, based on the results shown in Table 3, the total volume of aerosols in the range of 40–20,000 nm can be estimated in zones, the boundaries of which are determined by the place of aerosol generation and the place of measurement. With a constant consumption of the solution by the generator at the level of 1 cm^3^/min., the total volume of aerosols, depending on the variant A/Variant B, would be: 1.2 × 10^−2^ / 4.7 × 10^−4^ cm^3^ (zone: 0–10 m), 9.1 × 10^−3^ / 2.0 × 10^−3^ cm^3^ (zone: 0–25 m), and 2.5 × 10^−2^ / 5.6 × 10^−3^ cm^3^ (zone: 0–50 m). On the other side, the total potential volume of liquid in these zones can be evaluated while taking the air velocity and the corresponding retention time of produced liquid inside the zones into account. According to the estimation, only 0.2 to 3% of the total amount of liquid volume that is produced by the generator, which remains inside the zone, is in a form of aerosols in the range of 40–20,000 nm. It is only a rough approximation with such an assumption as a homogeneous concentration of aerosols inside the zone. In fact, the concentration of aerosols and their size inside the zone depends on many factors, such as temperature, humidity, pressure, and distance from the generator.

It is assumed that the breathing rate for workers is equal to 1.2 m^3^/h [28]. This means that, during 8-h work, the air volume intake by the respiratory system will be 9.6 m^3^. During each individual cough, 6.7 mg of saliva is expelled [26]. Assuming that the coughs occur once a minute, the volume of aerosols can be estimated in the range of 40–20,000 nm, which could enter the respiratory system of workers who are at distances of 10 m, 25 m, and 50 m from the source. These are the following volumes: 4.4 × 10^−6^ cm^3^ (10 m), 1.4 × 10^−6^ cm^3^ (25 m), 1.9 × 10^−6^ cm^3^ (50 m) for the Variant A, and 1.8 × 10^−7^ cm^3^ (10 m), 3.1 × 10^−7^ cm^3^ (25 m), 4.2 × 10^−7^ cm^3^ (50 m) for the Variant B. The SARS-CoV-2 RNA levels in saliva one week after symptom onset can range from around 10⁴ to 10^8^ viruses/cm^3^ [29], and other scientists report the peak concentration even from 10^6^–10^11^ viruses/cm^3^ [30]. When taking the given intake volumes of aerosols into account, this would correspond to a low intake probability of SARS-CoV-2 RNA (10⁴ viruses/cm^3^) to intake by the inhalation of 4.4 × 10^5^ viruses during one working shift if their concentration in saliva would be extremely high (10^11^ viruses/cm^3^).

The number of miners in Poland whose work is related to one specific place where hard coal is mined in an underground mine is of approximately 200–250 during one working shift. However, the working places are not close each together.

**Figure 10:**
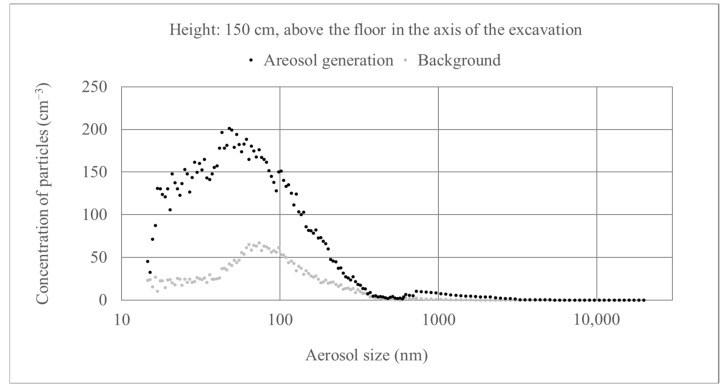
Particle size distribution before and during operation of the aerosol generator. Generator efficiency: 17 cm^3^ of solution/min.

Figure 10 presents the differences in size distribution before and during the operation of the aerosol generator. The concentration increases mainly relate to ultrafine and fine aerosols. Each of these distributions is an average of three 3-min. measurements. Larger fluctuations are seen in the case of aerosol generation, which may be caused by non-rhythmic operation of the unit and changing solution consumption rate.

## 4. Conclusions

Based on the results that were obtained in the whole cross-section of the excavation, the greatest background disturbances were observed close to the aerosol generator for aerosols larger than 2.5 µm. However, as the distance increased, the increase in aerosol concentration in the whole section of the excavation at the height of 150 cm became increasingly uniform.

Under conditions of higher temperature and lower humidity (Variant B), the aerosols decreased very quickly in size and a few coarse and supercoarse particles reached a position located 10 m away.

The concentration of additional aerosols in the class of ultrafine and fine aerosols increased with the distance in Variant A and B and the concentration of coarse particles decreased. Even when assuming solution consumption at a small level of 1 cm^3^/min., the number of additional aerosols was several hundred particles in one cubic centimeter of air. The final risk assessment should be made by virologists, even with the pessimistic assessment that each of them is a virus carrier.

Despite the small number of coarse and supercoarse aerosols, the additional volume that was brought by these aerosols during generator operation was predominant under lower temperature and humidity conditions and it varied between 60% and 90% (Variant A). At higher temperature and lower humidity, fine particles had a greater share in this range, which was 50–80% (Variant B). However, when assessing the risk, one should be very careful, because the distribution of coronavirus on these aerosols may be different, not being entirely proportional to the volume of the aerosols. Indeed, the formation of the resultant decomposition is a complex mechanism that depends on many factors, such as the size distribution of ambient aerosols, the size distribution of the aerosols containing the viruses themselves, and environmental conditions. A more accurate recognition of these mechanisms would require the use of appropriate markers in the size of 40–200 nm, which would not be destroyed in water.

Aerosols in still air can persist for a long time before they fall under gravity. For example, 10 µm particles will fall from a height of 150 cm for approximately 10 min., but, for an aerosol of 200–300 nm, it is about 140 h. This means that, in the case of forced ventilation, aerosols can travel many kilometers, in practice all the way in the underground excavation to the exhaust shaft. The results indicate that it is difficult to count on their significant loss due to inertia or diffusion. Even gravitational deposition may prove to be ineffective due to the reduction of particle size with high water content through evaporation. Such a mechanism will be particularly noticeable in conditions of low humidity and higher temperatures. Therefore, it can be concluded that a significant reduction in the concentration of additional aerosols is possible due to the supply of other air currents and ventilation to the ventilation network, rather than the loss of aerosols due to mechanisms, such as gravity, inertia, or diffusion.

Many researchers have noted the possibility of the virus transmission by aerosols [11,12,13,14,15], and the US Mine Safety and Health Administration (MSHA) has confirmed its importance, which recommends that miners wear appropriate masks to prevent respiratory droplets or aerosols from reaching others [31]. Based on the results of the study [28,29,30], how many viruses can be potentially absorbed by the respiratory system during a working day was estimated when there is one infected person in the team of employees and 6.7 mg/min. of saliva is secreted . This strongly depends on its concentration in saliva and, when considering the respiration rate, it can vary from a low intake probability by inhalation when the density is 10^4^ viruses/cm^3^ to about 4.4–10^5^ for a density of 10^11^ viruses/cm^3^, even when the distance from the source is 50m, which is largely influenced by forced air circulation. Therefore, the wearing of the masks is of great importance because the approved means of protection of the upper respiratory tracts, i.e., FFP2 with a leakage of 0.06, can reduce virus intake by inhalation by a factor of approximately 0.0036 (transmission coefficient of aerosols through two masks).

## Figures and Tables

**Table 1:** Environmental conditions during measurements (T—temperature, p—atmospheric pressure, and RH—relative humidity).

Variant	Distance (m)	T (°C)	p (mbar)	RH (%)	Average Air Speed (m/s)		
					Left Side Wall	Middle	Right Side Wall
A	0	12	978	89	−	−	−
A	10	12	978	96	0.43	0.35	0.38
A	25	12	978	98	0.43	0.35	0.38
A	50	12	978	95	0.43	0.35	0.38
A	Ventilation gallery	12	981	95	1.84	2.00	2.05
B	0	23	981	50	-	-	-
B	10	17	981	77	0.42	0.70	0.50
B	25	17	981	76	0.42	0.70	0.50
B	50	16	981	82	0.42	0.70	0.50

**Table 2:** Concentration of additional aerosols averaged over the left and right side walls and in the axis of the excavation at 150 cm, assuming that the consumption of solution by the aerosol generator is 1 cm^3^/min. The values for the left side wall, center and right side wall of the excavation are given in brackets.

**Variant**	**Distance (m)**	**Average Concentration of Additional Aerosols (cm^–3^) Within the Various Size Ranges (nm)**			
		40–20,000	40–100	100–2500	2500–20,000
A	10	158 (77|314| 84)	122 (48|240|79)	35 (29|72|5]	0.6 (0.2|1.5|0.1)
A	25	262 (349|168|267)	167 (230|94|177)	94 (119|74|90)	0.3 (0.4|0.3|0.3)
A	50	315 (363|316|266)	209 (248|206|172)	106 (114|110|94)	0.5 (0.6|0.5|0.4)
B	10	140 (159|138|122)	90 (101|92|77)	50 (58|46|45)	< 0.1 (< 0.1|< 0.1|< 0.1)
B	25	281 (513|176|154)	191 (340|130|102)	90 (173|45|52)	< 0.1 (< 0.1|< 0.1|< 0.1)
B	50	370 (386|409|315)	243 (254|270|205)	127 (133|139|111)	< 0.1 (< 0.1|< 0.1|< 0.1)

**Table 3:** The volume of additional aerosols in air volume and percentage terms at 150 cm averaged for the left and right side walls and in the axis of the excavation assuming that the consumption of solution by the aerosol generator is 1 cm^3^/min. The values for the left side wall, center and right side wall of the excavation are given in brackets.

**Variant**	**Distance** **(m)**	**Average Volume Concentration of Additional Aerosols in µm^3^/cm^3^ Within the Various Size Ranges (nm)**			
		40–20,000	40–100	100–2500	2500–20,000
		µm^3^/cm^3^	(%)	(%)	(%)
A	10	68 (18|176|9)	0.1 (0.0|0.0|0.2)	12 (19|8|10)	88 (81|92|90)
A	25	21 (27|20|18)	0.1 (0.1|0.1|0.2)	37 (38|39|34)	63 (62|61|66)
A	50	29 (33|31|23)	0.1 (0.1|0.1|0.1)	35 (36|33|35)	65 (64|67|65)
B	10	2.8 (4.6|2.7|1.1)	0.8 (0.4|0.5|1.3)	47 (36|56|48)	53 (64|44|50)
B	25	4.8 (7.5|4.5|2.4)	0.7 (0.7|0.5|0.7)	78 (87|68|81)	21 (12|35|39)
B	50	6.6 (5.2|8.0|6.6)	0.6 (0.8|0.6|0.5)	71 (87|64|61)	29 (12|35|39)

## Data Availability

The data presented in this study are available on request from the corresponding author.

## Sample Availability

The following samples are available from the authors: solution for aerosol generation.
